# Validation of Kiswahili version of WHOQOL-HIV BREF questionnaire among people living with HIV/AIDS in Tanzania- a cross sectional study

**DOI:** 10.11604/pamj.2023.44.95.36007

**Published:** 2023-02-16

**Authors:** Nuru Abdallah Kondo, Tumbwene Mwansisya, Eric Aghan, Godfrey Mutashambara Rwegerera, Riaz Ratansi

**Affiliations:** 1 Department of Family Medicine, Aga Khan University, Dar es Salaam, Tanzania,; 2 Department of Psychology and Psychiatry Neuro-imaging, School of Nursing and Midwifery, Aga Khan University, Tanzania,; 3 Department of Internal Medicine, University of Botswana, Gaborone, Botswana

**Keywords:** WHOQOL-HIV BREF, HIV, living with HIV, quality of life, validity, reliability

## Abstract

**Introduction:**

World Health Organization (WHO) has developed HIV specific quality of life tool called World Health Organization Quality of Life brief questionnaire in HIV population (WHOQOL-HIV BREF) for assessing the quality of life of people living with HIV/AIDS (PLWHA). Despite its sound validity and reliability from several studies, the developers recommend it to be validated in different cultures to assess its psychometric properties before its adoption. The study aimed at evaluating the validity and reliability of the Kiswahili version of the WHOQOL-HIV BREF questionnaire in Tanzania among people living with HIV/AIDS.

**Methods:**

a cross-sectional study with 103 participants recruited via systematic random sampling. The internal consistency of the questionnaire was assessed by the Cronbach alpha coefficient. Validity of the WHOQOL-HIV BREF was assessed through analysis of construct, concurrent, convergent and discriminant validity. The model performance was assessed by exploratory and confirmatory factor analysis.

**Results:**

the mean age of the participants was 40.5 ± 9.702 years. The internal consistency of the items of the Kiswahili version of WHOQOL-HIV BREF shows Cronbach's alpha values of 0.89-0.90 (p < 0.001). Analysis of test-retest reliability showed a statistically significant Intra-class correlation (ICC) of 0.91 - 0.92 (p < 0.001). The spiritual and physical domains were highly discriminated from the rest of the domains (Psychological, Environmental, Social and Independent domain).

**Conclusion:**

Kiswahili WHOQOL-HIV BREF tool was found to have good validity and reliability among Tanzanian people living with HIV/AIDS. These findings provide support for the use of this tool in assessing the quality of life in Tanzania.

## Introduction

HIV/AIDS is a global epidemic with an estimated 37.7 million people worldwide affected in 2020 [1]. As of June 2021, 28.2 million people were accessing antiretroviral therapy, up from 7.8 million in 2010 [1]. Sub-Saharan Africa (SSA) bears the greatest burden of HIV infections, with 71% of all global estimates [2]. The introduction of highly active antiretroviral therapy (HAART) in 1996 and its widespread availability have succeeded in prolonging life by reducing mortality and morbidity related to AIDS [1]. AIDS-related deaths have been reduced by 64% since the peak. In 2020, around 680 000 people died from AIDS-related illnesses worldwide, compared to 1.9 million people in 2004 [1]. The achievements attributed to the benefit of HAART have radically changed the natural history of HIV disease from the previously fatal disease status to the current chronic disease status. Currently, people living with HIV/AIDS (PLWHA) on HAART with higher adherence to treatment are expected to have a life expectancy closer or similar to that of the general population [1]. Tanzania is among the sub-Saharan Africa countries with a high HIV burden. It has an overall HIV prevalence of 4.6% in adults in 2018, with females being the most affected. Among PLWHA who know their status in Tanzania; 90.9% are on treatment and 87.7% of those on treatment are virally suppressed [3]. PLWHA naturally face different challenges that impair their quality of life. These challenges are attributed to HIV disease itself, stigmatization, HAART adverse effects and secondary comorbidities. Thus, the assessment of the quality of life is an important goal in the care of PLWHA [4-8].

Quality of life (QOL) is a broad multidimensional concept which addresses the general sense of wellbeing. Despite various attempts to describe the concept of QOL, there is no universal definition [9]. To unravel such limitation, WHO proposed a definition of QOL that could serve as a starting point to develop a thorough measure for assessing QOL. The World Health Organization Quality of Life (WHOQOL) Group defined QOL as an individuals´ perception of their position in life in the context of the culture and value systems in which they live, and in relation to their goals, expectations, standards and concerns [10]. Quality of Life in HIV disease has been widely studied for the last three decades among different populations of PLWHA including; older patients, pediatric populations, women and military patients [11]. Miner and colleagues have shown that PLWHA have lower QOL compared to HIV-negatives people [11]. Living with a chronic condition, relationships problems, psychosocial issues, discrimination and potentially adverse effect of HAART are believed to be a possible explanation for this decline in quality of life [11]. Various factors have been proven to strongly affect the QOL of PLWHA both in positive and negative aspects [12]. The factors include; - satisfaction with the health system, patient adherence to treatment, age, sex, social support, depression, long standing illness, functional disability, symptoms severity and level of CD4+ lymphocytes counts [12]. Depression and anxiety are the most prominent psychological issues encountered among PLWHA and are linked to a significant decline in QOL [11,13]. These issues were also noted in the pre-ART era and were linked to the intense fear of losing the two powerful life experiences (sex and life). The HAART has significantly reduced the fear of death and improve the sex life of individuals, however, the depression rates in PLWHA are still higher as compared to HIV seronegative counterparts. Experts on this field of QOL recommend the screening of mental health to enhance the quality of life regardless of the underlying cause of depression and/or anxiety [13]. Under-diagnosis or mismanagement of these mental health problems will decrease adherence to medication and facilitate the progression to AIDS and subsequently lowering the survival rate and quality of life [11].

HIV/AIDS associated symptoms such as fatigue, energy loss, nausea, insomnia, chronic diarrhoea, and chronic pain can decrease adherence to medication, limit social interaction, increase dependency level and, hence lower the quality of life [14]. In summary, all domains of life (physical, social, psychological and functional) can be affected by HIV disease resulting to lower the quality of life of an individual. These findings demonstrated the need for health professionals caring for PLWHA to screen and treat appropriately. Despite the existence of the many questionnaires, there is no ideal cross-cultural questionnaire to screen the QOL of PLWHA in routine clinics. Several tools have been used to assess the QOL of PLWHA. Majority of these tools were developed in a single culture and some of them used a poorly constructed models that omit the key aspects of QOL [15]. Other tools were too long to administer and hence cumbersome in a routine busy clinic. This raises many questions regarding how best to assess the QOL in a clinical environment and how such an instrument would influence the current routine care of PLWHA patients [16]. Based on the WHO definition, WHO quality of life team in 2002 in the effort of filling this gap developed an HIV specific questionnaire to be used in a routine clinic called World Health Organization Quality of life brief questionnaire in HIV population (WHOQOL-HIV BREF). The questionnaire was developed from the initial WHOQOL 100 generic version, which was standardized for QOL assessment in the general population [17]. Researchers from six countries with different cultures pooled together and agreed on the important aspects of QOL and concerns among PLWHA. The advanced methodology used in the development of WHOQO-HIV BREF has enabled the language versions to have greater semantic and conceptual equivalence between different cultures [17]. This means the original WHOQOL-HIV BREF questionnaire when translated into other languages, the loss of similar vocabulary meaning and/or construct across the two versions is very low.

Several studies have been done to assess the validity and reliability of WHOQOL-HIV BREF. The results have demonstrated overall sound validity and excellent reliability [18-20]. In a systematic review of HIV QOL generic and specific tools done by Cooper *et al*., WHOQOL-HIV BREF was shown to be the most valid cross-cultural tool [21]. It is therefore recommended as a good choice for international assessment of quality of life in PLWHA. Despite the good preliminary result of WHOQOL-HIV BREF from existing studies, Cooper *et al*. recommended further validation studies on different cultures [21]. WHOQOL team acknowledge as well further validation studies of WHOQOL-HIV BREF in new cultures, especially in SSA where the burden of HIV is high and there is a dearth of data on quality of life assessment among PLWHA [17]. Although widely used, WHOQOL-HIV BREF has not been validated in Tanzania settings. For a tool to be effective, the translation to the local language is recommended [17]. Kiswahili is the national language and is used by 95% of the Tanzanians. The aim of this study was to assess the validity and reliability of the Kiswahili version of WHOQOL-HIV BREF among PLWHA in Tanzania.

## Methods

**Study design, setting and participants:** this was a cross-sectional study conducted among PLWHA attended at an outpatient clinic at Mnazi Mmoja centre, in Dar es Salaam, Tanzania from 1^st^ September 2019 to 30^th^ November 2019. The centre has approximately 12,000 PLWHA attending for care and is one of the largest centres for HIV services in Tanzania. The inclusion criteria for this study were participants with a diagnosis of HIV infection for at least six months, age 18 and above and those who could read and write. PLWHA with psychiatric conditions, dementia, and other cognitive diseases were excluded from this study.

**Sample size calculation:** in this study, we opted to use the prevalence formula as well as multiplication methods used in validation studies to calculate the sample size. In Tanzania, the HIV prevalence is 4.5%, using a power of 80%, alpha error of 5%, and attrition rate of 10%, a sample size (N) of 73 was obtained, as calculated by using the formula for cross-sectional design [22]. According to the multiplication method, the questionnaire has 31 questions, using a range of 3 and an attrition rate of 10%, a sample size of 102 was obtained. Hence the sample size of 102 was used in this study as it is larger compared to the one obtained from the prevalence formula. Systematic random sampling was used to reduce selection bias. A sampling interval (n) of five was employed to recruit eligible participants according to the attendance registry book.

**Data collection tool:** the WHOQOL-HIV BREF questionnaire was used for data collection. It contains 31 items that have five extra items HIV related. It is shorter to administer; it only takes 10 minutes to complete the questionnaire [23]. It is a self-administered questionnaire. The WHOQOL-HIV BREF items are grouped into six domains, which are physical, psychological, level of independence, social relationships, environment and spirituality/religion/personal beliefs domains. Each domain has different facets which were rated on 5 points Likert scale; where 1 indicated a negative perception and 5 indicated a positive perception. Original English WHOQOL-HIV BREF questionnaire was used to assist with scoring and coding.

**Translation protocol:** we sought permission from the World Health Organization office to translate the WHOQOL-HIV BREF. The three-phase process of translation of WHOQOL-HIV BREF original English version was done as per WHO guideline, where the English version was translated into Kiswahili using bilingual translators with a medical background [24]. The Kiswahili version obtained was then translated back to English by an independent translator, and then compared with the original English version. The Kiswahili questionnaire obtained was piloted to assess its feasibility.

**Pre-testing of the Kiswahili WHOQOL-HIV BREF research tool:** the feasibility and reliability of the final Kiswahili questionnaire were tested on 17 randomly selected HIV patients (one-third of the total sample size) from the Aga Khan HIV clinic. This pilot study result demonstrated excellent reliability (Cronbach a of 0.90). Findings on the semantic and item equivalence were used to modify the questions flow before conducting an actual study. This pilot sample was excluded in actual data collection to avoid contamination.

**Data collection procedure:** the participants were asked to fill out the WHOQOL-HIV BREF Kiswahili questionnaire as they presented for their routine clinic. Four weeks later, the same participants were asked to fill out the same questionnaire. This duration was determined for test-retest reliability as it is long enough to avoid recall bias. Questionnaires whose completeness was less than 80% were excluded from the analysis.

### Data analysis

Data entries and analyses of results were done using the Statistical Package for Social Sciences (SPSS, version 25.0) software and the analysis of moment structure (AMOS), version 26.0. Data screening revealed 6 % of missing data, which was acceptable to run the analysis. The characteristic distribution of the variables was assessed by employing Kurtosis and the central limit theorem, which showed a normal distribution of the data. This information was used to apply the parametric statistical tests in this study. Descriptive statistics of the participants were determined, categorical and numerical data were presented by frequencies and means. The validity of the Kiswahili version of the WHOQOL-HIV BREF questionnaire was evaluated using a variety of methods. The translation validity for both face and content validity were assessed through the meticulous translation protocol of the WHO. The construct validity of WHOQOL-HIV BREF was assessed using the exploratory factor analysis (EFA) [25]. AMOS software in the SPSS was used to confirm the factor structure of the WHOQOL-HIV BREF from the exploratory analysis [26]. The goodness of fit was evaluated using comparative fit index (CFI), X2, root mean square error of approximation (RMSEA), and Probability value (P-value). A Confirmatory Factor Index (CFI) value of > 0.90, RMSEA value of < 0.08 and P-value > 0.05 indicate an adequate model fit. Concurrent validity was assessed using the Pearson correlation r due to the normal distribution of the data set.

We correlated the WHOQOL-HIV BREF domains with three self-evaluated general health measures provided in the WHOQOL-HIV BREF questionnaire. We hypothesized the individual with a high perceived score of quality of life would have high scores in domains of the WHOQOL-HIV BREF questionnaire. The correlation coefficients above 0.3 are recommended when comparing two questionnaires that measure the same construct. Convergent validity was assessed using the Pearson r correlation of the items with their domains. The discriminant validity was assessed using correlation coefficients and average variance extracted (AVE). The reliability of the WHOQOL-HIV BREF was assessed using an intra-class coefficient for consistency using Cronbach´s alpha. The test-retest reliability was assessed using an intra-class coefficient of agreement using Pearson´s r. The duration for retesting was four weeks, which was sufficient to prevent participants from remembering the previous responses and was short enough for major health changes to occur.

**Ethical considerations:** this study was reviewed and approved by the Aga Khan University Research committee (AKU-RC) and The Aga Khan University Ethics Review Committee (AKU-ERC) before study commencement. District Medical research board of Ilala Municipal gave permission for data collection from the Mnazi Mmoja hospital. All recruited participants signed an informed written consent form and were free to withdraw consent at any stage of the study. All considerations of the Declaration of Helsinki were taken into account.

## Results

**Figure 1:**
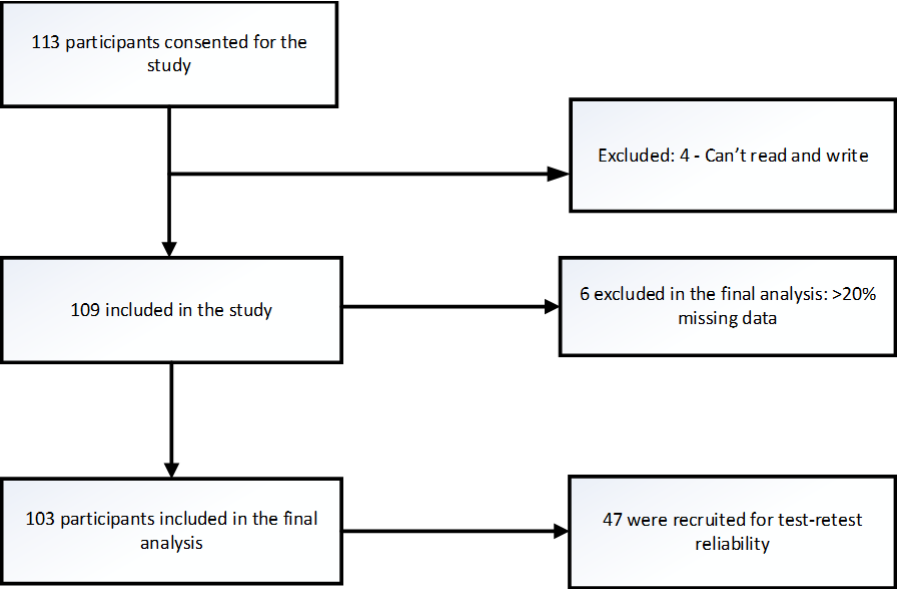
flow diagram of study participants

A total of 103 participants were enrolled in phase one of the study. Forty-eight out of 103 participants were later included in phase two of the study to assess for test-retest reliability of the Kiswahili version of WHOQOL-HIV BREF. A flow diagram indicating the flow of participants for this study is shown in Figure 1.

**Socio-demographic characteristics:** the mean age (standard deviation) of the participants was 40.5 (9.702) with two-thirds being between 45 years and below (67.0%). The majority of the participants were women (66, 64.1 %), whereas (42, 40.8%) of the participant were married. Of the 103 recruited participants, 71 (68.9%) reported being infected through the heterosexual route. The majority of participants (85, 82.5%) were asymptomatic for HIV with meantime (standard deviation) since diagnosis being 8.8 (6.27) years. Socio-demographic and HIV -related characteristics of the participants are shown in Table 1.

**Table 1:** descriptive analysis of the baseline characteristics of the study population

Variable	Frequency	Percentage (%)
**Gender**		
Male	37	35.9
Female	66	64.1
**Age (years)**		
18-25	5	4.9
26-35	29	28.2
36-45	33	32.0
46-55	26	25.2
56-65	6	5.8
≥66	1	1.0
**Marital status**		
Single	31	30.1
Married	42	40.8
Separated/Divorced	14	13.6
Widow	16	15.5
**Level of education**		
Primary	48	46.6
Secondary	41	39.8
Tertiary	14	13.6
**Mode of HIV transmission**		
Sex with male/female	71	68.9
Injecting drugs	2	1.9
Blood products	6	5.8
Others	24	23.3
**HIV symptom status**		
Asymptomatic	85	82.5
Symptomatic	13	12.6
AIDS	5	4.9

### Score distribution of WHOQOL-HIV BREF

The mean (standard deviation) scores distribution of the six domains for 103 participants ranged from 10.17 (3.18) to 22.20 (4.86). Across domains, the environmental domain had the highest mean score (22.2 ±4.86). The overall floor and ceiling effect values for items with minimum and maximum scores were 2.2% and 6.0% respectively (values above 20% are considered significant). On the other hand, the following items had a very high ceiling effect; physical pain (33.9%), HIV symptoms (33.9%), self-esteem (30.1%), non-medical dependence (28.2%), mobility/get around (27.2%), daily activities capacity (24.3%), health services availability (33.0%), stigma (40.8%), fear of the future (39.8%) and death worries (58.3%). A high floor effect was observed in the item measuring sexual activity (24.3%). Skewed distribution was not observed for any of the items.

**Reliability:** analysis of the 31 items showed a Cronbach alpha coefficient of 0.89 - 0.90. This result indicates that the Kiswahili version of WHOQOL-HIV BREF has acceptable internal consistency. The test-retest reliability showed a statistically significant Intra-class correlation for all items. The test-retest values were good, with the ICC ranging from 0.91-0.92 (p < 0.001). Table 2 shows the distribution of inter-class correlations for individual facets.

**Table 2:** distribution of Intra-class correlation and internal consistency of the Kiswahili version of WHOQOL-HIV-BREF facets

Domains	Cronbach Alpha (n=103)	Intra-class correlation (n=47)	P-value
Purposefulness	0.892	0.916	<0.001
Pleasure/Enjoyment	0.890	0.912	<0.001
Stigma	0.898	0.919	<0.001
Fear of the Future	0.896	0.918	<0.001
Death worries	0.899	0.918	<0.001
Attentiveness	0.895	0.914	<0.001
Secure feelings	0.893	0.911	<0.001
Environmental safety	0.891	0.911	<0.001
Energy	0.889	0.911	<0.001
Body Appearance	0.892	0.912	<0.001
Financial stability	0.889	0.912	<0.001
Social involvement	0.892	0.912	<0.001
Informative tools	0.889	0.912	<0.001
Leisure/Recreation	0.892	0.912	<0.001
Get around	0.890	0.912	<0.001
Sleep	0.891	0.911	<0.001
Daily activities	0.890	0.910	<0.001
Work capacity	0.891	0.911	<0.001
Self-love	0.891	0.912	<0.001
Personal relationships	0.892	0.912	<0.001
Sex life	0.896	0.916	<0.001
Friends/Relative support	0.888	0.911	<0.001
Living condition	0.889	0.910	<0.001
Health services	0.892	0.911	<0.001
Transport system	0.890	0.914	<0.001
Negative feelings	0.895	0.911	<0.001

### Validity

**Construct validity:** the six domains of WHOQOL-HIV BREF model were assessed by confirmatory factor analysis (CFA) using the AMOS software to examine whether it explains the relationships among domains and facets. The majority of the WHOQOL-HIV BREF items produced substantial factor loadings and analysis of model fit produced an acceptable fit to the model (X^2^= 658.319, df =362). The analysis of model performance was further analyzed using Root Mean Square Error of Approximation (RMSEA = 0.09) showed good performance. However, CFI of 0.68 and p-value of < 0.001 were below the acceptable range (recommended value: CFI = >0.90, P-value > 0.05).

**Concurrent validity:** WHOQOL-HIV BREF had a moderate correlation with three self-evaluated general questions/items (the overall quality of life, general health perception, and self-perceived health status). It was found that scores of four domains (psychological, social, environment and level of independence) were positively correlated with the three self-evaluated general questions (all with Pearson´s correlation coefficient within the domains of statistical significance and above 0.3 that is recommended for evaluating concurrent validity). Table 3 shows the correlation between each domain score with the three self-evaluated general questions.

**Table 3:** correlation between Kiswahili version of WHOQOL-HIV BREF and general health measures

Domain	Self-evaluated QOL	Self-evaluated general health	General health
Physical	0.13	-0.04	-0.07
Psychological	0.37***	0.22**	0.52***
Environmental	0.29**	0.20**	0.32**
Social	0.37***	0.38**	0.34**
Level of independence	0.44***	0.21**	0.34**
Spiritual	0.03	-0.60	-0.15

**p-value 0.01 to < 0.05; ***p-value < 0.001

**Convergent validity:** convergent validity was determined by the correlation between items and their respective domains. All items showed moderate to strong correlations with their respective domain and r coefficients ranged from 0.243 to 0.762 (p < 0.01). The highest correlations of items were seen in the social domain where r coefficients ranged from 0.62 -0.76. The overall convergent validity was good. Table 4 shows the correlation between items and their respective domains.

**Table 4:** the Pearson correlation between items and their respective domains

Domain	Item-domain correlation
**Physical**	
Physical pain	-0.485**
HIV symptoms	-0.596**
Energy	-0.477**
Sleep	0.480**
**Social**	
Sexual activities	0.616**
Personal relationship	0.708**
Friends support	0.762**
Social inclusion	0.656**
**Environmental**	
Secure feelings	0.615**
Environmental safety	0.638**
Financial stability	0.665**
Informative tools	0.703**
Leisure	0.669**
Living condition	0.538**
Health services	0.563**
Transport system	0.669**
**Spiritual**	
Stigma	-0.741**
Fear of the Future	-0.627**
Death worries	-0.579**
Enjoyment	-0.058**
**Independent**	
Medical dependence	-0.340**
Mobility	0.676**
Daily activities	0.641**
Work Capacity	0.665**
**Psychological**	
Attentiveness	0.608**
Purposefulness	0.409**
Body appearance	0.568**
Negative feelings	-0.249**
Self-Love	0.667**

** p-value 0.01 to < 0.05

**Discriminant validity:** concerning discriminant validity, spiritual and physical domains were highly discriminated from the rest of the domains. Their correlation coefficients were lower compared to their squared root of average variance extracted (AVE). The other four domains were poorly discriminated, with their correlation coefficients greater than their squared root of AVE. These results concluded that the discriminant validity was satisfactory when compared to the three domains found in the exploratory factor analysis of Kiswahili WHOQOL-HIV BREF.

## Discussion

The results of this study suggested that the Kiswahili version of WHOQOL-HIV BREF is a valid and reliable instrument for the evaluation of the quality of life in PLWHA. In general, the internal consistency and test-retest reliability of the Kiswahili version of WHOQOL-HIV BREF was excellent. The construct validity of the questionnaire measured by major types of validity evidence (translational, concurrent, convergent, and discriminant) provides strong valid results supporting its use in quality of life screening of PLWHA in our setting. The mean age (Standard deviation) of our study participants was 40.5 ± 9.7 years, which is similar to the Georgia and Portugal validation studies [27,28].

The vast majority of the participants were female and the heterosexual route was the predominant mode of HIV transmission; this is similar to the Ethiopian study [19]. The predominance of the female could be explained by the inability to negotiate safe sex among African women and the cultural aspects of early marriages. In other studies, the majority of the participants were male and the homosexual route was the predominant mode of HIV transmission [18,28,29].

Descriptive analysis of the scores showed a ceiling effect on some of the items. It was noted that the scores on these items indicated the most favorable circumstance for the respondents and it can be attributed to the religious belief around the items in question. This ceiling pattern did not affect the normality of the distribution for these items that warrant the use of the non-parametrical method. Floor effect was detected in the item measuring a sexual activity and this is due to the cultural difficulty in revealing one´s sexual practice. Other studies also encountered floor and ceiling effects in different items according to their cultural context [18,20,27].

Regarding the reliability of the Kiswahili version of WHOQOL-HIV BREF, our study demonstrated an excellent internal consistency of the tool. Test-retest reliability of the WHOQOL-HIV BREF also was excellent, hence strengthening the reliability of the study questionnaire. These findings were similar to the majority of the studies that were conducted in other settings [19,20,27-29]. The Kiswahili WHOQOL-HIV BREF questionnaire was found to be valid. This was deducted through meticulous analysis of validity parameters that showed a good construct validity. These results were similar to other studies previously done using language translated versions of the WHOQOL-HIV BREF questionnaire [20,27-29].

Quality of life measured by an individual through three self-evaluated general questions showed a high correlation with the scores in the domains except for the physical and spiritual domains. This reflects the convergence of the construct towards the quality of life outcome. The low correlation of the physical and spiritual domains has been also reported in Malaysia and Taiwan studies [20,29]. The two possible explanations for these findings are; first, the presence of overlapping constructs between physical and spiritual which failed to discriminate the items leading to different interpretation/perceptions. Second, studies have indicated that religion and culture can have an influence on the lifestyle and shape the experiences of illness, pain, and end-of-life care. The majority of Tanzanians are likely to have religious beliefs that are associated with poor medical seeking behaviours [20,29,30]. The convergent validity of Kiswahili WHOQOL-HIV BREF was found to be excellent. The social domain had the highest correlation among the six domains. This highlight the role of a good social support system in enhancing the quality of life of an individual. The discriminant validity was satisfactory, a finding that was observed in another previous study [30].

### Limitations

This is the first study on validation of Kiswahili WHOQOL-HIV BREF in Tanzania. The results need to be interpreted with caution due to several limitations. The sensitivity to change of the Kiswahili WHOQOL-HIV BREF was not assessed due to the cross-sectional design used. This design is not useful for the assessment of the sensitivity to changes in the clinical status of people living with HIV/AIDS.

## Conclusion

The WHOQOL-HIV BREF questionnaire revealed excellent reliability and validity among Tanzanian people living with HIV/AIDS. These findings provide evidence to support the use of the WHOQOL-HIV BREF as a tool of QOL screening among PLWHA in Tanzania. Data on quality of life can be obtained using this questionnaire and can help us to target different needs that are arising in PLWHA populations. This can help in resources allocation, and planning future interventional studies that may result in improvement of quality of life among PLWHA.

### What is known about this topic

- People living with HIV/AIDS (PLWHA) face different challenges that impair their quality of life;
- Assessment of the quality of life is an important goal in the care of HIV-infected individuals;
- The advanced methodology used in the development of WHOQO-HIV BREF has enabled the language versions to have greater semantic and conceptual equivalence between different cultures.

### What this study adds

- Kiswahili WHOQOL-HIV BREF tool was found to have good validity and reliability among people living with HIV/AIDS, supporting its use to assess quality of life among PLWHA in Tanzania;
- The social domain had the highest correlation among the six domains of Kiswahili WHOQOL-HIV BREF tool, highlighting the role of a good social support system in enhancing the quality of life of PLWHA in our setting.
